# The Schistosomiasis Consortium for Operational Research and Evaluation Rapid Answers Project: Systematic Reviews and Meta-Analysis to Provide Policy Recommendations Based on Available Evidence

**DOI:** 10.4269/ajtmh.19-0806

**Published:** 2020-05-12

**Authors:** Charles H. King, David Bertsch, Gisele N. Andrade, Michael Burnim, Amara E. Ezeamama, Sue Binder, Daniel G. Colley

**Affiliations:** 1 Center for Global Health and Diseases, Case Western Reserve University, Cleveland, Ohio;; 2 Schistosomiasis Consortium for Operational Research and Evaluation, Center for Tropical and Emerging Global Diseases, University of Georgia, Athens, Georgia;; 3 Escola de Enfermagem, Universidade Federal de Minas Gerais, Belo Horizonte, Brazil;; 4 Department of Psychiatry, College of Osteopathic Medicine, Michigan State University, East Lansing, Michigan;; 5 Department of Epidemiology & Biostatistics, University of Georgia, Athens, Georgia;; 6 Department of Microbiology, University of Georgia, Athens, Georgia

## Abstract

The Schistosomiasis Consortium for Operational Research and Evaluation (SCORE) was established in late 2008 to conduct operational research to inform global health practices related to the control and elimination of schistosomiasis. The greatest part of the SCORE investment has been in multiyear, long-term efforts, including cluster-randomized trials of gaining and sustaining control of schistosomiasis, trials on elimination of schistosomiasis, and diagnostic test development and evaluation. In the course of planning and conducting SCORE studies, critical questions were raised that could be answered relatively quickly by collecting, collating, and synthesizing existing data. Through its Rapid Answers Project (RAP), the SCORE conducted seven systematic reviews, including four associated meta-analyses, on issues related to screening for schistosomiasis, enhancing mass drug administration, treatment impacts, and the efficacy of snail control for prevention of human schistosomiasis. This article summarizes the findings of the seven RAP reports and provides links to the studies and their supporting information.

## INTRODUCTION

The Schistosomiasis Consortium for Operational Research and Evaluation (SCORE) was funded by the Bill & Melinda Gates Foundation in 2008 to conduct research that would inform programmatic decision-making for schistosomiasis control.^1^ Although most of the SCORE investment was focused on large, multiyear efforts, the SCORE has also conducted smaller, focused work to answer questions that arose during planning for and conducting the larger studies.

Starting in 2009, the SCORE convened meetings of experts and program stakeholders to better define study questions and protocols for its major areas of work. During the resulting discussions, several questions arose that prompted quick literature searches. It became clear that there were a number of relevant questions for which studies had already been published but for which a synthesis of the available information was not available, and hence, “evidence-based” policy was lacking. For example, during an early planning meeting to design the studies of gaining and sustaining control of schistosomiasis,^2^ the question was raised whether a program could reliably use urine dipsticks to assess local prevalence of *Schistosoma haematobium*, especially at the time of intake eligibility screening, for determining whether a potential study community met a prevalence cutoff criterion for participation in SCORE randomized trials. A related question was whether dipstick performance in a community would be impacted by previous mass drug administration with praziquantel. A quick Web search during the meeting identified several studies that might include relevant data, but these had not been assembled and evaluated in a way that provided a clear summary estimate and an assessment of the strength of the evidence.

By the middle of 2009, the SCORE Rapid Answers Project (RAP) had begun. The purpose of RAP was to conduct systematic reviews of the literature to answer questions of importance for study design or to inform program practices, with a focus on questions for which a reasonable amount of data was available. The first RAP was published in 2011, and a total of seven RAPs have been completed.^3–9^

The first three questions addressed by RAPs were as follows:

• **RAP 1**: What is the impact of double treatment (two doses close together) versus single dosing for treatment of *Schistosoma mansoni* and *S. haematobium*?^3^
• **RAP 2**: How well do urine dipsticks perform for assessing prevalence of *S. haematobium* in low-prevalence or previously treated areas?^4^
• **RAP 3:** Do adults living in areas endemic for *S. haematobium* get reinfected after treatment, and, if so, at what rate?^5^

As the SCORE’s modeling work expanded and work began on developing the protocol for the elimination study in Zanzibar, questions about the efficacy of snail control became a priority.^10^ Before the introduction of safe oral drug therapy, snail control had been the key intervention for reducing schistosomiasis.^11^ However, by the time the SCORE project began in 2009, the practice of controlling snails to reduce *Schistosoma* transmission had become much less common in African settings.

• **RAP 4:** This work included a historical review of the perceived pros and cons related to snail control and a list of the technical inputs required for delivering snail control.^11^ It also produced a formal meta-analysis of field studies on the impact of molluscicide-based control programs on the risk of local human infection.^6^
• **RAP 5:** As the SCORE’s large intervention studies were implemented, concerns were raised about treatment coverage^12^ and how to reach children who were not in school. This resulted in a systematic review published as RAP 5.^7^
• **RAP 6:** Specific questions related to policy support for morbidity control were addressed in RAP 6 in regard to how treatment-related reductions in *Schistosoma* infection intensity actually translate into reduction in infection-related morbidities.^8^
• **RAP 7:** This work examined the impact of chronic schistosome infection on cognitive function and educational performance in children.^9^

All RAP studies were published in the peer-review literature, with the exception of RAP 3, for which there were little good quality data available. Brief two-page summaries of the RAPs have been developed and are available online as Supplemental Files 1–7 for this article.

## APPROACH TO INFORMATION COLLECTION AND SYNTHESIS

The paucity of research evidence for clinical and public health decision-making is a definite element of the “neglect” of neglected tropical diseases. In some cases, this is due to the absence of quality research data. In other cases, it has been due to difficulties in determining the sum of available evidence on a particular topic. The field of systematic review and meta-analysis of data across similar studies is a discipline that emerged just before the beginning of the SCORE project.^13,14^ We quickly realized that this approach had the potential to provide answers within a reasonable period of time to inform policy-related questions that had been previously studied in more than one location but without any global synthesis to provide generalizable recommendations.

We saw that meta-analysis techniques had the potential to clarify the significance of past studies—Neglected Tropical Disease studies have often been small (hence often underpowered statistically), focused on only a single location and underfunded in terms of data acquisition, testing, and statistical analysis. Meta-analysis allows for enhancement of study size through weighted inclusion of results from multiple studies. This pooling approach allows for detection of significant effects of infection that may be statistically small in effect size, but, nevertheless, can be clinically highly significant. For example, in many small individual studies, the magnitude of *Schistosoma* infection effects on health outcomes such as anemia, undernutrition, exercise intolerance, or cognitive impairment were often too subtle to be called “statistically significant” in that particular study’s analysis. The interpretation was then that there was “no adverse effect” from schistosomiasis and that the infection, for most people, was “asymptomatic” and, therefore, benign.^15^ The use of meta-analysis has now challenged this viewpoint and has allowed for identification of the multiple functional disabilities that are significantly related to *Schistosoma* infections. Early onset in preschool years, the chronicity of infection, and the cumulative impact of multiple waves of worm infection can now be tested for association with these “non-classic” forms of schistosomiasis-related morbidity.

## METHODS FOR SYSTEMATIC REVIEW AND META-ANALYSIS

Although meta-analysis is a very useful tool, it requires intensive detective work to uncover available evidence. It also requires specialized analysis and adherence to a prespecified protocol that outlines the project’s primary study question, search strategies, data curation, analytic approaches, and plans for publication and data sharing.^16^ This strengthens reproducibility of results and can allow for relatively rapid updating of analysis as new studies become available for inclusion, with reanalysis of quantitative estimates. Steps in the performance of a systematic review and meta-analysis are summarized in Table 1.

**Table 1:** Steps in performing a systematic review and meta-analysis

Stage	Task
Step 1	Formulate the research question.
Step 2	Develop the a priori study protocol and work schedule and register and publish it online at the International Prospective Register of Systematic Reviews (PROSPERO register).
Step 3	Begin the organized search and archiving of available literature, using translation where necessary to include publications not in English (this was important for schistosomiasis because of the large number of publications in Chinese, Portuguese, and French). Searches must also seek for “gray literature,” which are data resources in official reports or other publications not found in scientific journals. Researchers should similarly look for materials such as book chapters that may contain relevant data but are not indexed in online systems. The project should request and keep on file scans of articles not immediately available in electronic versions.
Step 4	After exhaustive searching by topic, titles, and abstracts of recovered materials are reviewed to determine their likelihood of having usable data for meta-analysis. Promising articles are then read in full and data reviewed (with checks for non-duplication, human focus, and target population relevance) in sufficient detail to be included in the analysis.
Step 5	Data extracted are curated in a searchable database including relevant information on study design, populations, locations, interventions, etc.
Step 6	Summary statistics are generated for the study outcomes of interest. Assessment of heterogeneity across studies is then performed, and where appropriate, random effects modeling is used to provide the summary estimates of effect sizes in outcomes. There should be sensitivity analysis by subgroup, including assessment of risk of study bias.
Step 7	Presentation and publication of results are required. Updates to protocol registration are carried out to indicate completion and archiving of data used in the meta-analysis.

There were controversies and recognized limitations to our meta-analysis approach. Essentially, a meta-analysis is an observational study that is wholly dependent on the availability of previous studies. There is risk of “publication bias” in summing the results of published studies. This is because of past unwillingness of many journals to publish negative results. There are within-discipline debates about inclusion of “low-quality” data from observational cross-sectional and case-control studies and from nonrandomized control trials. These carry risk of bias in study outcomes due to site and subject selection, but they also reflect conditions highly relevant to program operations, that is, the factors that are present outside a controlled study, which can significantly affect the outcomes of an intervention.^17–19^

The time needed to perform each fully comprehensive systematic review and meta-analysis ultimately took over 2 years, which was too long for truly “rapid” answers to some of our questions. Some workers in the field have suggested ignoring older literature and non-English publications to create rapid publication of “Knowledge to Action” summaries of latest studies.^20^ For schistosomiasis, this approach was unlikely to work well because many of the best schistosomiasis studies from the twentieth century would have, thus, been ignored in such a circumscribed review.

There continues to be an ongoing tension between careful summarization of available evidence versus the wish to have more conclusive findings for evidence-based decision-making. Given the status of schistosomiasis research so far, we must use a “preponderance of the evidence” for much of our guidance. Nevertheless, systematic compilation of the evidence and weighted summation of the data comprise an important step forward from our past reliance on non-systematic/nonquantitative reviews and/or expert opinion. Even if meta-analysis is not technically possible, performance of a systematic review can offer the best chance for unbiased answers in the appraisal of specific interventions.^3,5,7^ In our RAP program, we found that four of the seven systematic reviews provided multi-study data of sufficient quality for formal quantitative meta-analysis, allowing for elaboration of generalized summary estimates of the targeted study outcomes.^4,6,8,9^

## SUMMARIES OF SCORE RAPID ANSWERS PROJECT (RAP) STUDY QUESTIONS AND ANSWERS

**RAP 1. What is the impact of double treatment for *S. mansoni* and *S. haematobium***, **when a second praziquantel dosing is given 2–8 weeks after initial treatment?****^3^**

*Key findings:* This project’s systematic review found 11 studies (in 10 articles) that met the study criteria for inclusion. In Africa, on average, repeated dosing 2–8 weeks apart appears to offer particular advantages in the treatment of *S. mansoni*, the cause of intestinal schistosomiasis. However, repeated dosing has less consistent impact for *S. haematobium*, the cause of urogential schistosomiasis. Our Markov model cost-effectiveness projections suggest significant incremental benefits from double dosing in terms of 1) limiting a person’s total years spent infected and 2) limiting the number of years they spend with heavy infections, with consequent improvements in quality of life. In addition, our model suggests that double dosing is cost-effective in controlling infection-associated morbidity. Results of this RAP were later influential in the design of the SCORE study on interruption of seasonal transmission of *S. haematobium* in northern Côte d’Ivoire.^21,22^

**RAP 2. How well do urine dipsticks perform for assessing prevalence of *S. haematobium***, **including low prevalence and previously treated areas?****^4^**

*Key findings:* Seventy-one reports, containing data on 95 separate surveys, were included in this study. Our meta-analysis indicated that dipsticks retain their validity as a diagnostic tool, even when eggs in the urine are scarce, or become so, after a round of therapy. Because of the presence of “egg-negative” schistosomiasis in *S. haematobium* transmission zones, the diagnostic specificity of dipstick heme diagnosis is likely to be greater than previously believed. Our meta-analysis indicated that commercial dipsticks, designed for rapid detection of heme in the urine, continue to provide an effective proxy for detection of active *S. haematobium* infections in disease-endemic areas. The systematic evidence from this study allowed the SCORE to choose dipstick screening for more rapid eligibility screening of endemic communities in Niger for the enrolment in the *S. haematobium* control projects that were performed there.^2^

**RAP 3. Do adults in endemic areas get reinfected with *S. haematobium* following curative drug treatment?****^5^**

*Key findings:* This systematic review found 14 studies from across Africa that looked for evidence of adult reinfection with *S. haematobium* following curative drug treatment. These studies indicated that following successful cure by praziquantel, adults in endemic areas can experience *S. haematobium* reinfection at rates varying from zero to 1.5% per month. Although this rate is much lower than that typically found among children, periodic retreatment of adults may be necessary to limit disease and mitigate their role in maintaining transmission. Evidence from this RAP led to the inclusion of adults in the community-based praziquantel delivery in a 5-year SCORE trial on approaches to elimination of *S. haematobium* in Zanzibar.^22,23^

**RAP 4. How effective is chemical mollusciciding in reducing snail numbers and in reducing local *Schistosoma* infection risk?****^6^**

*Key findings:* Our meta-analysis of 63 studies performed between 1953 and 1981 catalogued a wide variety of snail control treatments and schedules. Among studies reporting on human infections, we found that snail control reduced local human schistosomiasis prevalence and incidence of infection in most, but not all, locations. Estimates from the aggregated studies indicate that snail control (alone) typically reduced new infections by 64%, and local prevalence declined over a period of years. This decline was accelerated and more profound (84% reductions) if drug treatment was also made available. Early results of this RAP aided in the design of molluscicide interventions in the elimination trials in both Zanzibar^24^ and Côte d’Ivoire,^21^ described in Campbell et al.^22^

**RAP 5. How do different mass drug administration delivery methods compare in terms of achieving high coverage of enrolled and non-enrolled school-aged children (SAC)? What other individual**, **community**, **or programmatic factors are associated with high- or low-coverage rates?****^7^**

*Key findings:* For this RAP, outcomes data from 22 selected studies were evaluated. The studies indicated that combined community-wide and school-based delivery achieves the highest median coverage of SAC, followed by community-only delivery, then school-only delivery. The WHO guidelines recommend at least 75% coverage, which was achieved by all included studies that used combined distribution, but not by all studies using community-only or school-only distribution. Across all included studies, non-enrolled children had lower MDA coverage overall than enrolled children, and school-based delivery had the lowest coverage of non-enrolled children compared with other delivery methods. Lack of knowledge about therapy, fear of side effects, and poor motivation of drug distributers significantly contributed to gaps in MDA coverage.

**RAP 6. Does treatment of *Schistosoma* infection translate into reduced odds of infection-related morbidity? If so**, **by how much?****^8^**

*Key findings:* This project identified 71 studies in 64 publications that met our inclusion criteria for this topic. Meta-regression indicated that posttreatment reductions in egg burden are significantly correlated with decreased morbidity. In particular, larger egg reduction rates (ERRs), which indicate acute reductions in worm burden, are associated with reversal of most acute pathology. More advanced chronic pathologies appear less responsive to single rounds of treatment, even with adequate ERRs, and multiple rounds of treatment may be necessary to improve those outcomes. Factors affecting the magnitude of morbidity reductions included *Schistosoma* species, population studied, age and infection status of study participants, and how long after treatment follow-up occurred. The quantitative findings of this RAP study are now being incorporated in new cost-effectiveness analyses of approaches to schistosomiasis morbidity control.

**RAP 7. How does *Schistosoma* infection affect childhood cognitive function and school performance?****^9^**

*Key findings:* Systematic review identified 30 reports that met our inclusion criteria for this project. Meta-analysis revealed that *Schistosoma* infection (versus noninfection) or schistosomiasis nontreatment in placebo-controlled trials was significantly associated with educational, learning, and memory deficits in SAC. Early treatment of children in *Schistosoma*-endemic regions could potentially prevent or mitigate these deficits. Results from this most recent RAP study are expected to provoke a policy-level reconsideration of the importance of early childhood treatment for all forms of schistosomiasis.^25^

## SUMMARY

With an average publication delay of 1.5–2 years, we, like others,^20^ discovered that a thorough search for evidence is a painstaking and time-consuming process. The SCORE RAPs proved to be not so “rapid” after all. Nevertheless, preliminary results of each systematic review helped guide individual SCORE projects in choosing the most promising options in study implementation. Full quantitative meta-analysis and final publication took longer, but they were ultimately rewarding in terms of the additional knowledge and perspectives gained about each topic.

Systematic reviews aim to provide a nonselective and an as-neutral-as-possible assessment of the available published and non-published data relevant to a given management problem. Performance of this type of focused review has now become standard practice in the development and promulgation of disease intervention guidelines by the WHO and other advisory groups.^26^ The techniques of the related numerical meta-analysis have the potential to develop more generalizable summary estimates of treatment impacts. By means of combining data from similar studies, they also have the potential to establish small but clinically relevant effects as statistically significant, when smaller (possibly underpowered) individual studies did not.^13^ Combined with in-depth sensitivity analysis, systematic review and meta-analysis results can yield a more convincing picture of where, when, and how health-related interventions can be useful.

The techniques of systematic review and meta-analysis continue to evolve, meaning that revision and updates of our findings are likely to occur. Greater online availability of published scientific articles (and their underlying data), as well as data-sharing by national and regional control programs, strengthens the quality of the evidence base used for systematic reviews. Readers interested in updating the SCORE RAP reviews can consult the search strategies and supporting information published with each review and should feel free to contact the corresponding authors for each project.

## Supplemental files

Supplemental materials
