# Supplementary material for: The Schistosomiasis Consortium for Operational Research and Evaluation Rapid Answers Project: Systematic Reviews and Meta-Analysis to Provide Policy Recommendations Based on Available Evidence
Source: Am J Trop Med Hyg. 2020 May 12;103(1 Suppl):92–6. doi: 10.4269/ajtmh.19-0806 (PMC7351305; doi:10.4269/ajtmh.19-0806)
Supplement: Supplementary file 1 [file tpmd190806.SD1.pdf]

# SCORE Rapid Answers Project:
# The Impact of Double Treatment for *S. mansoni* and *S. haematobium*

## SCORE Research Question

***Background:*** Controversy persists about the optimal approach to drug-based control of schistosomiasis in high-risk communities. In a systematic review of published studies, we examined evidence for incremental benefits from a second praziquantel dosing, given 2 to 8 weeks after an initial dose, in *Schistosoma*-endemic areas of Africa.[1]

***RAP Question***: What is the impact of double treatment (2 doses close together) for *S. mansoni* and *S. haematobium*?

**The figure below shows the impact of single vs. double praziquantel dosing for 'cure'** of *Schistosoma* in high-risk African communities. The upper panel shows efficacy of one- and two-dose regimens for treatment of *S. mansoni* according to the initial pre-treatment infection prevalence of study participants. The lower panel shows the relative efficacy of one- vs. two-dose treatment of *S. haematobium*.

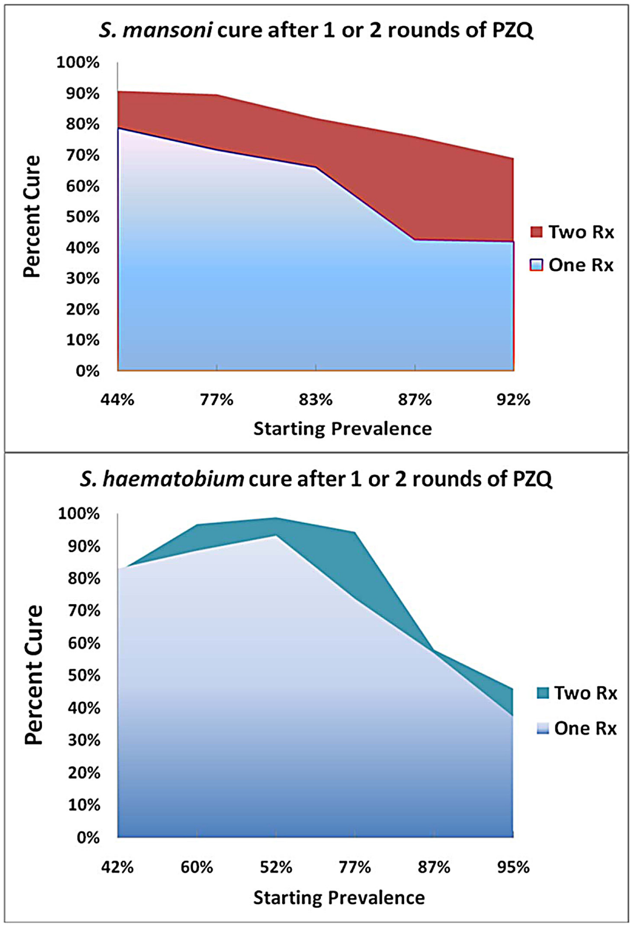


doi:10.1371/journal.pntd.0001321.g002

## Schistosomiasis Treatment

Schistosomiasis remains a significant health burden for many parts of the world, particularly where health resources are most limited. At least 239 million people had active *Schistosoma* infection in 2009, 85% lived in sub-Saharan Africa, where an estimated 150,000 deaths/year were attributable to schistosomiasis. Although praziquantel has been used for treatment for nearly 30 years, mass treatement has become much more widely available in recent years due to large drug donations, improved transportation infrastructure in some places, and other factors.

Our study was a systematic review of population-based studies that compared single- vs. double-dose praziquantel treatment of *Schistosoma mansoni* or *S. haematobium* in high-risk locations in Africa. We also examined the projected costs and long-term impacts of these alternative strategies in a simulated 'problem' community setting where schistosomiasis is highly endemic.

## What We Found

Several studies have shown that a second dose of praziquantel, given 2 - 8 weeks after the first dose, can improve cure rates and reduce the intensity of remaining infections in population-based programs.

Our systematic review of published research found that in Africa, on average, such repeated dosing appears to offer particular advantages in the treatment of *S. mansoni*, the cause of intestinal schistosomiasis, but has less consistent impact after double dosing for *S. haematobium*, the cause of urogential schistosomiasis.

**Our projections suggest incremental benefits from double dosing** in terms of i) limiting a person's total years spent infected and ii) limiting the number of years they spend with heavy infections, with consequent improvements in quality of life, and, in addition, they indicate that double dosing is cost-effective in controlling infection-associated morbidity.

Schistosomiasis Consortium for Operational Research & Evaluation
CTEGD, University of Georgia, Athens, GA 30602
phone: 706-542-3334 fax: 706-542-3582 score@uga.edu
http://score.uga.edu

# Double Treatment for *S. mansoni* and *S. haematobium*

## At a Glance: Study Selelction Process

Potentially relevant studies indentified and screened for retrieval (n=55) → Studies retrieved for more detailed evaluation (n=49) → Potentially appropriate studies to be included in the meta-analysis (n=41) → Studies included in the meta-analysis (n=16) → Studies with usable information, (n=11) from 10 published papers

- 6 studies excluded as overviews and reviews. These were used only for reference to more relevant studies
- 8 studies excluded: 2 excluded as information presented not pertinent to number of treatments; 1 excluded because time frame between treatment and follow-up not included; 4 excluded as morbidity of infection the focus without regard to follow-up; 1 excluded as 2nd follow-up occurred 3-5 years after 1st treatment
- 25 studies excluded: 10 excluded as subjects not treated at 2nd follow-up (1 treatment); 14 excluded as information not collected after 2nd treatments; 1 excluded as subjects treated twice, but information reported only after 1st mass treatment
- 6 studies excluded based on the use of special subgroups, non-standard dosing, or prolonged interval between 1st and 2nd doses

## Results

Results of our systematic review of treatment outcomes suggest there were significant improvements to be gained in terms of *S. mansoni* infection outcomes by implementing a two-dose regimen in which patients were retreated 2-8 weeks after their initial PZQ dose. By contrast, a single-dose PZQ treatment was very nearly as effective as the more aggressive double-dose treatment of *S. haematobium* (see Figure on previous page).

The analysis also demonstrated that treating children or communities with two sequential doses of PZQ could be a cost-efffective treatment plan for areas with high initial prevalence of *S. mansoni* or *S. haematobium*, even in the face of ongoing transmission risk (see Table below).

**The table below shows the incremental cost-effectiveness of a two dose regimen** compared to a single dose regimen in a continuing community-wide mass drug campaign.

| Strategy | Life years spent infected by species[a] | Years spent with heavy infection | Cumulative lifetime cost[b] | Incremental Cost | Lifetime Egg-Years[b] | Lifetime QALYs[b] | Incremental cost-effectiveness[c] | | |
|---|---|---|---|---|---|---|---|---|---|
| | | | | | | | $ per infection year averted | $ per egg year averted | $ per QALY gained |
| Without treatment | 19.7 | 6.8 | $0.00 | – | 2973 | 26.96 | – | – | – |
| One dose per annual treatment, ages 5–55 | 9.1 Sm 7.4 Sh | 1.1 Sm 0.9 Sh | $23.01 | $23.01 | 657 Sm 529 Sh | 27.46 Sm 27.47 Sh | $2.17 Sm $1.87 Sh | $0.01 Sm $0.009 Sh | $47.90 Sm $45.58 Sh |
| Two doses per annual treatment, ages 5–55 | 5.6 Sm 5.7 Sh | 0.2 Sm 0.2 Sh | $46.03 | $23.02 | 223 Sm 229 Sh | 27.52 Sm 27.52 Sh | $6.58 Sm $13.54 Sh | $0.053 Sm $0.077 Sh | $291.07 Sm $432.76 Sh |

[a]Abbreviations: Sm, *Schistosoma mansoni* infection; Sh, *Schistosoma haematobium* infection; QALY, Quality-adjusted Life Year.
[b]Time discounted at 3% per annum.
[c]Relative to strategy in the row immediately above.
doi:10.1371/journal.pntd.0001321.t004

[1]Charles H. King, Stephanie K. Olbrych, Maragaret Soon, Mendel E. Singer, Jen Carter, Daniel G. Colley. 2011. Utility of Repeated Praziquantel Dosing in the Treatment of Schistosomiasis in High-Risk Communities in Africa: A Systematic Review. PLOS Negl Trop Dis 5(9): e1321. doi: 10-1371/journal.pntd.0001321
