# Supplementary material for: The Schistosomiasis Consortium for Operational Research and Evaluation Rapid Answers Project: Systematic Reviews and Meta-Analysis to Provide Policy Recommendations Based on Available Evidence
Source: Am J Trop Med Hyg. 2020 May 12;103(1 Suppl):92–6. doi: 10.4269/ajtmh.19-0806 (PMC7351305; doi:10.4269/ajtmh.19-0806)
Supplement: Supplementary file 2 [file tpmd190806.SD2.pdf]

SCORE Rapid Answers Project (RAP):
Urine Dipstick Diagnosis of *S. haematobium*, Including Low-Prevalence, Low-Intensity, and Previously-Treated Settings

SCORE Research Question

Background: Urine dipsticks for detection of microhematuria offer an inexpensive way to estimate *Schistosoma haematobium* infection prevalence. However, their performance in areas with lower prevalence or in populations that have been previously treated has not been evaluated systematically.[1]

RAP Question: How well do urine dipsticks perform for assessing prevalence of *S. haematobium*, including low-prevalence and previously treated areas?

At-a-Glance: Study Search and Selection

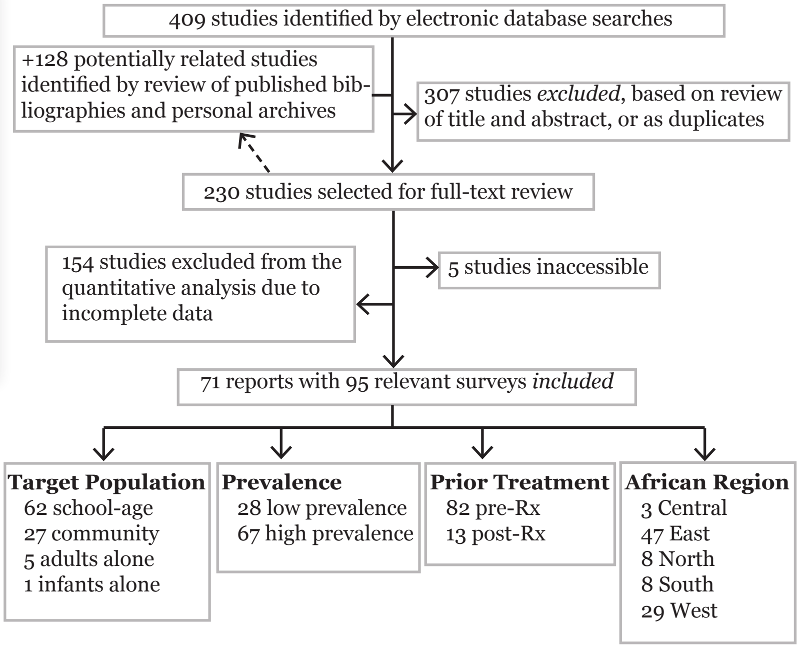

Urogenital Schistosomiasis

Schistosomiasis is a chronic disease caused by infection with trematode parasites of *Schistosoma* species. Urogenital schistosomiasis is caused by *S. haematobium*. *S. haematobium* colonizes veins in the pelvis and in and around the urinary tract and causes inflammation, ulceration, and bleeding into the urine.

S. haematobium is endemic in many areas of Africa and the Middle East that lack adequate sanitation and a safe water supply. Most control programs are based on drug administration targeted either to school-age children or to high risk communities at large.

The urinary dipstick for detection of hematuria has long been recommended as a relatively inexpensive and potentially accurate proxy for detection of *S. haematobium*. As with any diagnostic test, performance characteristics can vary with the underlying population prevalence of the targeted disease.

Our Approach

In our systematic review, we identified population surveys that compared dipstick diagnosis of *S. haematobium* infection to results based on urine egg counts for the same population. Final pooled summary estimates for dipstick sensitivity and specificity were calculated using hierarchical summary ROC (HSROC) regression. Sub-group analysis examined the impact of low (<20% egg-positive) vs. high (>20%) prevalence, light vs. heavy infections (as defined by authors of the included surveys), and whether the community had previously been treated.

Findings

Results of our analysis are summarized in the table on page 2. Overall, dipstick performance showed 81% sensitivity and 89% specificity for detection of egg-positive urines, and an estimated 82% sensitivity and 97% specificity for detection of active *S. haematobium* infection, as defined via a combination of dipstick and egg count results. This included possible 'egg-negative' schistosomiasis, wherein a screening urine test is negative, but the subject has active infection. Whereas dipsticks were less sensitive in detecting egg-positive urines among post-treatment populations and among subject sub-groups with lower intensity infections, evaluation of paired pre- and post-treatment studies did not show a consistent post-treatment effect on dipstick diagnostic performance.

Significant differences in dipstick diagnostic performance were noted based on the age range of survey subjects (school-age *vs.* community-wide). In addition, studies from North Africa were found to have significantly lower dipstick diagnostic performance, independent of the age of the subjects, community prevalence or treatment status, and other measured cofactors, suggesting a possible difference in risk for *S. haematobium*-associated morbidity between North African and sub-Saharan populations.

Important Themes from Our Meta-Analysis

- Urine dipsticks can provide an effective proxy for detecting *S. haematobium* infection, even in areas with low prevalence or in areas that have received treatment.
- Sensitivity of dipsticks appears to be lower in areas with low prevalence and higher among school-age children than in community-wide surveys.
- Dipstick performance varies from location to location, which was not fully explained by the available study characteristics.

Schistosomiasis Consortium for Operational Research and Evaluation
CTEGD, University of Georgia, Athens, GA 30602
phone: 706-542-3334 • fax: 706-542-3582 • score@uga.edu • http://score.uga.edu

Urine Dipstick Performance

Comparison of Dipstick Results Among Total Populations Versus the Subset of People with Light Infections

An ROC curve is a standard way to represent the performance data for a diagnostic test. The y-axis indicates reported sensitivity of the test, and the x-axis indicates specificity on an inverse scale. The better a test performs, the nearer it will be found to the upper left-hand corner of the plot.

The figure on the right summarizes dipstick sensitivity and specificity for detecting egg-positive infections in 25 studies from Africa that reported results for both the entire population (open black ellipses) and the subset of local subjects with light infections (solid red diamonds), with symbols proportional to study size. The long-dashed lines link each individual study's data points. The diamonds are always below the partner circles, indicating that sensitivity is always lower in the light infection subset.

Two smooth curves indicate our summary overall dipstick performance estimates derived by combining all studies in HSROC regression - for the total population performance, this is the black line; for the light-intensity subgroup, it is the lower red line.

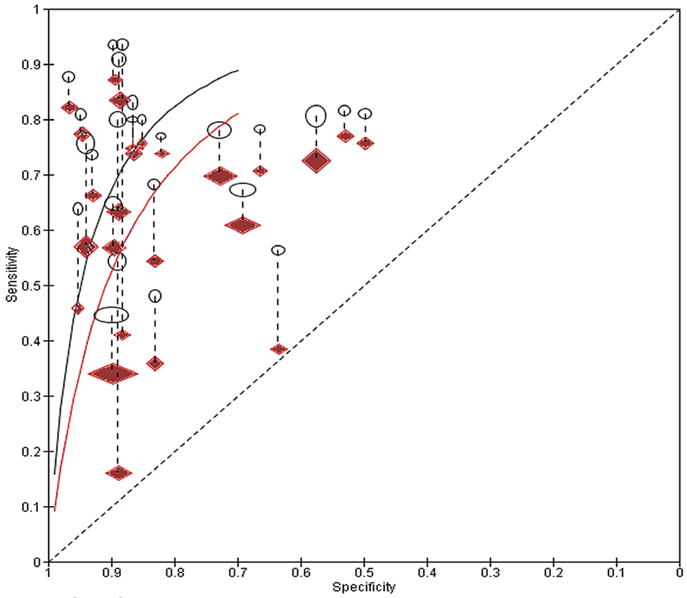

ROC curve comparing dipstick performance among people with low-intensity infections as compared to performance among the corresponding total population.

Conclusions

Despite the lack of a true gold standard for diagnosis of *S. haematobium* infection, our Bayesian analysis indicates that dipsticks retain their validity as a diagnostic tool, even when eggs in the urine are scarce, or become so, after a round of therapy. Because of the presence of 'egg-negative' schistosomiasis in *S. haematobium* transmission zones, the diagnostic specificity of dipstick heme diagnosis is likely to be greater than previously believed. Our meta-analysis indicates that commercial dipsticks, designed for rapid detection of heme in the urine, continue to provide an effective proxy for detection of active *S. haematobium* infections in disease-endemic areas. Reagent dipsticks will remain an efficient, practical, community-level diagnostic screening tool, even as programs reduce parasite prevalence and move towards elimination.

Summary of Overall and Subgroup Sensitivity and Specificity Estimates for Detection of Active Infection

Group/Subgroup	Sensitivity (95% CI)[a]	Specificity (95% CI)[a]
**All studies**	82% (80, 84%)	97% (95, 98%)
**High prevalence populations**	92% (89, 94%)	87% (83, 89%)
**Low prevalence populations[b]**	79% (73, 83%)	98% (95, 99%)
**Light intensity subgroup**	82% (74, 92%)	96% (91, 98%)
**Prior to treatment**	83% (80, 85%)	96% (94, 98%)
**Post treatment**	79% (68, 92%)	95% (84, 99%)

[a]Sensitivity and specificity for detection of active *S. haematobium* infection, as defined by a combination of egg count and hematuria status, assuming both tests are imperfect.

[b]Where community prevalence of *S. haematobium* is below 20%.

[1]King CH, Bertsch D (2013) Meta-analysis of Urine Heme Dipstick Diagnosis of *Schistosoma haematobium* Infection, Including Low-Prevalence and Previously-Treated Populations. PLoS Negl Trop Dis 7(9): e2431. doi:10.1371/journal.pntd.0002431
