# Supplementary material for: The Schistosomiasis Consortium for Operational Research and Evaluation Rapid Answers Project: Systematic Reviews and Meta-Analysis to Provide Policy Recommendations Based on Available Evidence
Source: Am J Trop Med Hyg. 2020 May 12;103(1 Suppl):92–6. doi: 10.4269/ajtmh.19-0806 (PMC7351305; doi:10.4269/ajtmh.19-0806)
Supplement: Supplementary file 3 [file tpmd190806.SD3.pdf]

SCORE Rapid Answers Project: Adults' Risk of Reinfection by *S. haematobium* in Endemic Communities in Africa

SCORE Research Question

Background: Mass drug administration (MDA) has become the standard approach for control and prevention of schistosomiasis. SCORE is researching whether community- or school-based drug delivery will be more effective in controlling *S. haematobium* infection and its related pathology. Experts disagreed about adults' risk for reinfection and, therefore, potential need for repeated adult treatments—

RAP Question: According to data from existing studies, do adults in endemic areas get reinfected with *S. haematobium* following curative drug treatment?

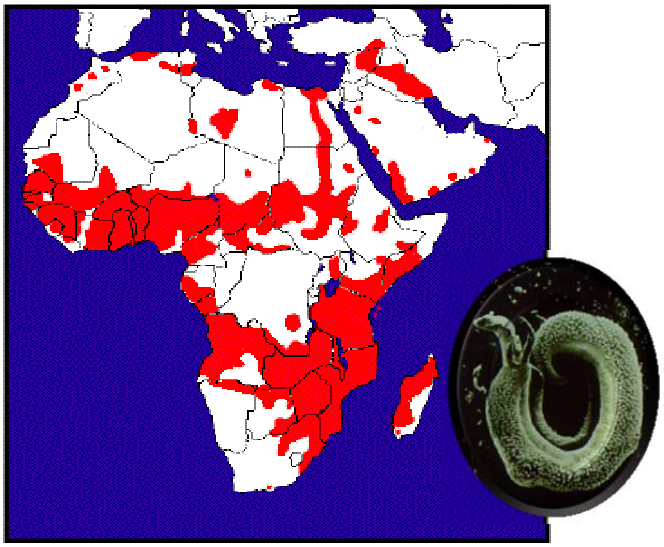

Figure 1: Areas at risk for *S. haematobium* are shown shaded in red

Schistosoma haematobium infection

S. haematobium is a multicellular (helminth) parasite of humans that causes chronic inflammatory disease of the kidneys, bladder, and reproductive organs. Infections are common throughout sub-Saharan Africa and the Middle East. In past years, *S. haematobium* transmission has been reported in 53 countries, with at least 111 million people requiring treatment in 2011. Transmitted via freshwater *Bulinus* species snails, *S. haematobium* is most commonly acquired during water contact activities such as fishing, fording, collecting water, cleaning, bathing, playing, and swimming. The most common symptom of the resulting urogenital schistosomiasis is painful bladder, often with blood in the urine. Persistent infections present with anemia, growth stunting, fibrosis of the bladder and/or ureter, infertility, kidney failure (due to outflow obstruction and bacterial superinfection), and bladder cancer.

Needs Assessment

S. haematobium can be controlled with the drug praziquantel. However, even if treatment is effective, and even if people develop partial immunity after infection, continued exposure to contaminated water can lead to reinfection. Reinfection has been well documented in children, but less is known about risk for reinfection among adults in endemic areas. This RAP 'knowledge-to-action' study compiled existing literature to determine whether reinfection by *S. haematobium* occurs at a significant rate after curative treatment of adults.

Adult reinfection with *S. haematobium*

At a Glance: Selection and Characteristics of Included Studies

1. LIVE IN ENDEMIC AREA
2. TESTED FOR INFECTION = EGGS IN URINE
3. PZQ DRUG TREATMENT
4. CURE ESTABLISHED AT FOLLOW UP 4-12 WEEKS LATER
5. CHECKED FOR *S. HAEMATOBIUM* REINFECTION 2.5-36 MONTHS LATER

Results

The literature summarized in this report is detailed in the accompanying spreadsheet, 'SCORE RAP 3 S haematobium reinfection in adults.xlsx'. The data indicate that adult reinfection with *S. haematobium* does occur, but with differing frequencies in different settings. Eleven of 14 studies documented adult reinfection after treatment, although 3 studies from Gabon, Senegal, and Egypt did not find any reinfection in older age groups. The graph below presents unadjusted per patient-month reinfection rates from the six studies that reported detailed data on cure rates and reinfection rates among treated adults.

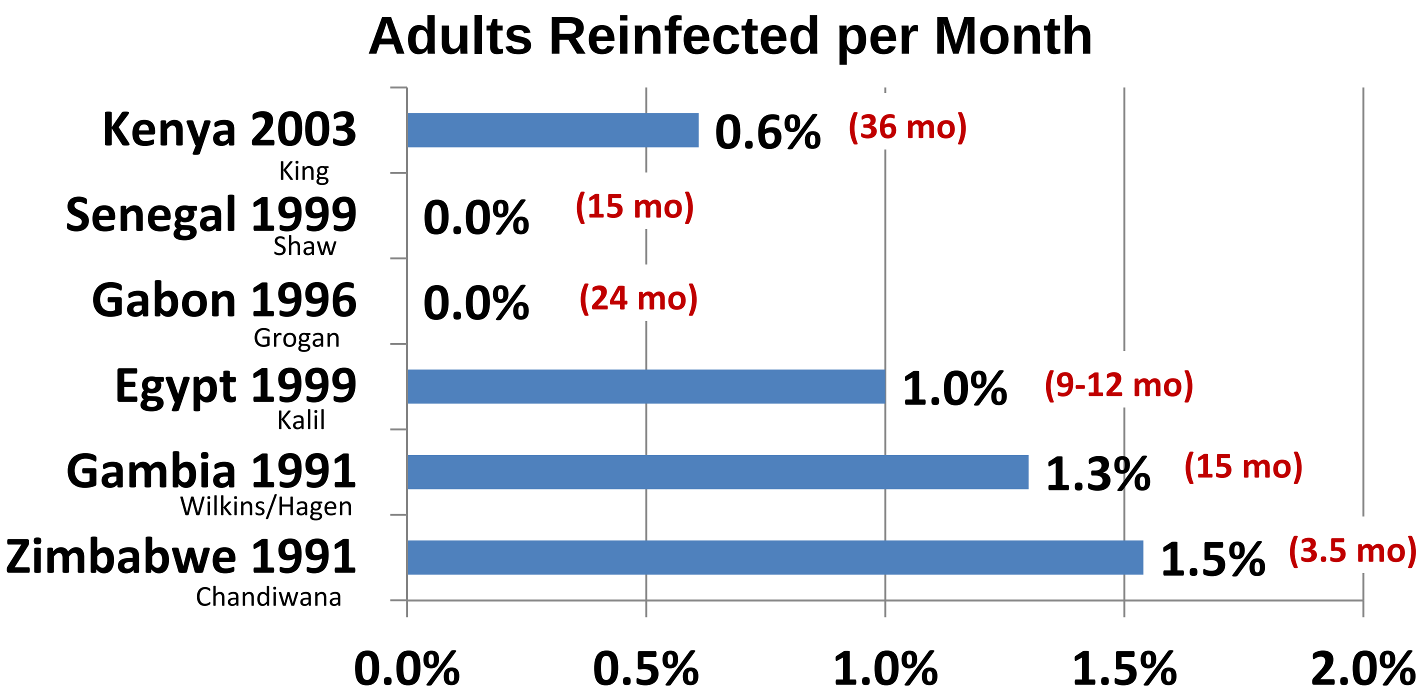

Figure 2: *S. haematobium* reinfection rates per patient-month of observation. The duration of follow-up is shown in parentheses. The paper from Egypt (Kalil, 1999) was clinic-based; the others were community-based. An additional seven studies provided evidence that adults do become reinfected, but rates could not be derived from those reports.

Summary

- Following successful cure by PZQ, adults do experience S. *haematobium* reinfection.
- While this rate is much lower than that typically found among children, periodic retreatment of adults may be necessary to limit disease and avert their role in continuing transmission.

Compiled and Written by Charles King, Stephen Dorner & Sophie Kerman
