# Supplementary material for: The Schistosomiasis Consortium for Operational Research and Evaluation Rapid Answers Project: Systematic Reviews and Meta-Analysis to Provide Policy Recommendations Based on Available Evidence
Source: Am J Trop Med Hyg. 2020 May 12;103(1 Suppl):92–6. doi: 10.4269/ajtmh.19-0806 (PMC7351305; doi:10.4269/ajtmh.19-0806)
Supplement: Supplementary file 4 [file tpmd190806.SD4.pdf]

SCORE Rapid Answers Project (RAP):

Effectiveness of Chemical Mollusciciding in Reducing Snail Numbers and Local *Schistosoma* Infection Risk

Background: Periodic drug treatments do not stop transmission of the parasite, which occurs when human urine or feces contaminate local water bodies with parasite eggs. These eggs infect intermediate host snails, which release the schistosome cercariae that are infectious for people. In this systematic review, we collated past experience of using chemically-mediated snail control for prevention of schistosomiasis.[1]

Question: How effective is chemical mollusciciding in reducing snail numbers and in reducing local *Schistosoma* infection risk?

In this Study

Our meta-analysis of 63 studies[1,2] (performed 1953-1981) catalogued a wide variety of snail control treatments and schedules. Among studies reporting on human infections, we found that snail control reduced local human schistosomiasis prevalence and incidence of infection in most, but not all locations. Estimates from the aggregated studies indicate that snail control (alone) typically reduced new infections by 64% and local prevalence declined over a period of years. This decline was accelerated and more profound (84% reductions) if drug treatment was also made available.

Table 1. Perceived advantages of using molluscicides

1	Direct interruption of snail-to-human transmission
2	Less need for intensive involvement of the community
3	A reasonable efficiency and cost of product
4	The simple equipment can also be used for the control of other vectors
5	While good supervision is essential, the methods of application are simple and do not require specialized operational schemes
6	Selection of foci for application can usually be based on the patterns of water use by the local population

Adapted from McCullough, et al. Bull WHO 58:681, 1980; and de Souza, Mem Inst Oswaldo Cruz 90:165, 1995.

doi:10.1371/journal.pntd.0003657.t001

Table 2. Perceived disadvantages of using molluscicides

1	Repeated reapplication is necessary, because snail eradication is not likely possible
2	Time demands of implementation and evaluation of control are greater than for MDA
3	The impact on *Schistosoma* infection and morbidity is delayed relative to drug therapy
4	Uniform dispersal and area coverage are difficult to achieve
5	The cost of labor is foremost when doing repeated treatments
6	Well-informed technical capacity is required to decide appropriate application
7	Collateral molluscicide effects on amphibians and fish must be openly addressed and effectively minimized to meet public concerns about safety and environmental impact

doi:10.1371/journal.pntd.0003657.t002

Schistosomiasis Consortium for Operational Research and Evaluation
CTEGD, University of Georgia, Athens, GA 30602
phone: 706-542-3334 • fax: 706-542-3582 • score@uga.edu • http://score.uga.edu

Snail Control

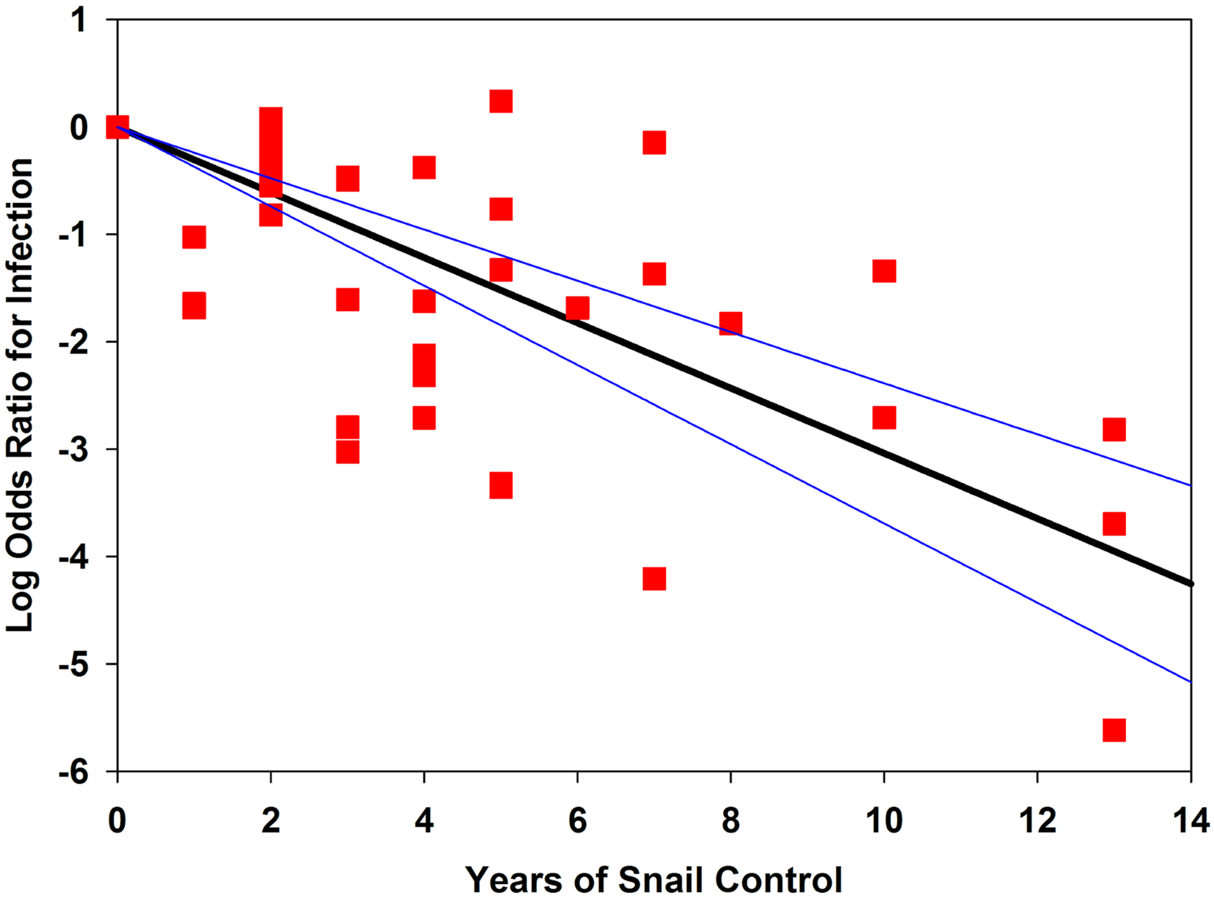

Fig 1. This meta-regression shows that after the first years of implementation, the duration of snail control efforts can have a big impact on *Schistosoma* infection prevalence. Each red squares represents a single study in the meta-analysis.[1] The x-axis indicates the years of snail control completed. The y-axis indicates the $\log_{10}$ (odds ratio) for infection, comparing post-control prevalence to pre-control prevalence. That is, the log odds ratio is an estimate of how much snail control appeared to reduce infection prevalence from the prevalance expected without snail control. A value of -3 is 1/10 the value of -2, and a value of -2 is 1/10 the value of -1, which reflects a 90% reduction in the odds of infection; so, the reductions seen after several years of snail control turned out to be quite large. The dark line is the best fitting regression line, and the thin lines are its 95% confidence limits.
doi:10.1371/journalpntd.0004290.g005

Findings

- Niclosamide use for snail control resulted in minimal risk to humans and the environment, although niclosamide is harmful to fish, amphibia, certain insect larvae, and, in higher doses, aquatic vegetation.
- Metered and very focal niclosamide administration at human water contact sites has the potential to provide the greatest impact on *Schistosoma* transmission with the least impact on local ecosystems.
- The included studies predominately indicate a positive effect of molluscciding in reducing *Schistosoma* transmission.
- These impacts are greatest after multiple years of snail control.
- Randomized comparison trials comparing mass drug administration with mass drug administration plus snail control are ongoing in Zanzibar and Cote d'Ivoire.

[1]King CH, Sutherland LJ, Bertsch D (2015). Systematic Review and Meta-analysis of the Impact of Chemical-Based Mollusciciding for Control of *Schistosoma mansoni* and *S. haematobium* Transmission. PLoS Negl Trop Dis 9(12):e0004290. doi:10.1371/journal.pntd.0004290
[2]King CH, Bertsch D (2015). Historical Perspective: Snail Control to Prevent Schistosomiasis. PLos Negl Trop Dis 9(4):e0003657. doi:10.1371/journal.pntd.0003657
