# Supplementary material for: The Schistosomiasis Consortium for Operational Research and Evaluation Rapid Answers Project: Systematic Reviews and Meta-Analysis to Provide Policy Recommendations Based on Available Evidence
Source: Am J Trop Med Hyg. 2020 May 12;103(1 Suppl):92–6. doi: 10.4269/ajtmh.19-0806 (PMC7351305; doi:10.4269/ajtmh.19-0806)
Supplement: Supplementary file 5 [file tpmd190806.SD5.pdf]

SCORE Rapid Answers Project (RAP):
Systematic Review of Coverage from Community-based, School-based, and Combined Delivery Strategies Targeting School-Aged Children for Mass Drug Administration for Schistosomiasis Control

Background
The mainstay of current schistosomiasis control is mass preventive chemotherapy of school-aged children (SAC) with praziquantel. Treatment is delivered through school-based, community-based, or combined school- and community-based strategies. To achieve a sustained effect on schistosomiasis prevalence and transmission, attaining very high treatment coverage among children is essential. It is similarly important to ensure that there are no persistently untreated subpopulations of children, a potential challenge for school-based programs in areas with low school attendance. Overall, this review sought to understand what program features, particularly which delivery method, led to the highest mass drug administration (MDA) coverage rates among school age children. We have also summarized authors' suggestions for improving MDA programs in the future.

Questions
1. How do different MDA delivery methods compare in terms of achieving high coverage of enrolled and non-enrolled SAC?
2. What other individual, community, or programmatic factors are associated with high or low coverage rates?

Methods
For this systematic review, we searched Medline for studies published before October 2015 that contained quantitative or qualitative data about MDA coverage rates for SAC being treated with praziquantel. Twenty-two studies, mostly observational, met criteria for inclusion in the systematic review. Fourteen had quantitative coverage information, and ten of the fourteen plus eight others had qualitative data about factors influencing coverage.

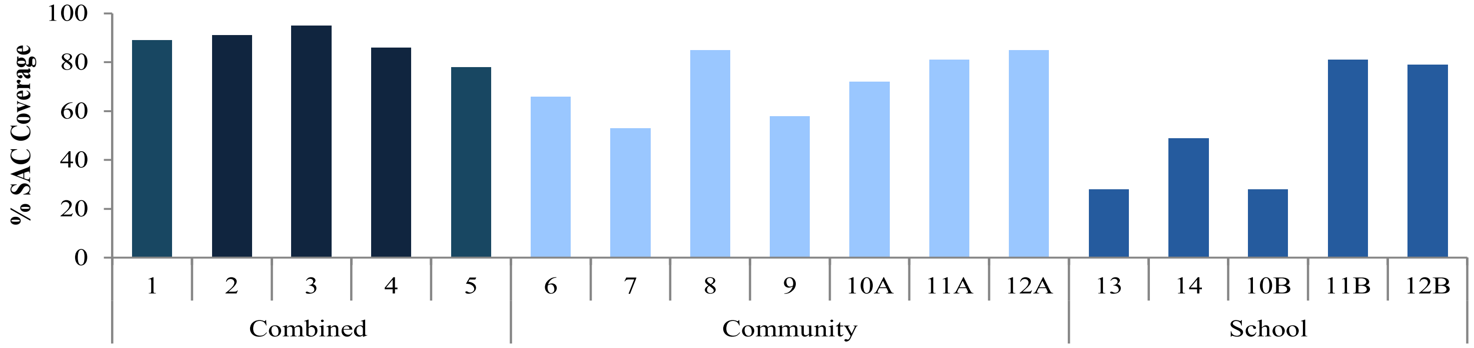

Fig 1. Praziquantel coverage rates, by delivery strategy. The reported MDA coverage rates for SAC in each quantitative study (1-14) are shown, grouped by delivery method (combined, community-based, and school-based). For the three studies comparing school- and community-based strategies (10, 11, and 12), community and school coverage rates are displayed in separate columns.

Key Results
- The highest coverage rates were achieved with combined community- and school-based delivery (range 78%-95%, median 89%). The second highest rates were achieved with community-only delivery, including the community arms of the comparative trials (range 53%-85%, median 72%). The lowest coverage rates were achieved with school-only delivery, including the school arms of the comparative trials (range 28%-81%, median 49%).
- Three studies reported on coverage for non-enrolled children. The one study comparing community- and school-based strategies to reach non-enrolled children had coverage rates of 82% and 58%, respectively. The other two studies only reported on combined strategies, and had coverage rates of 88% and 90%.
- Major factors affecting program success included fear of side effects, inadequate education about schistosomiasis, lack of incentives for drug distributors, and inequitable distribution among minority groups.

Schistosomiasis Consortium for Operational Research and Evaluation
CTEGD, University of Georgia, Athens, GA 30602
phone: 706-542-3334 • fax: 706-542-3582 • http://score.uga.edu

Improving MDA Coverage for School Age Children

Specific recommendations for increasing program effectiveness described in these articles include:

Goal	Strategies
Increase knowledge about the benefits of praziquantel	Include more education about praziquantel and schistosomiasis in drug distributor training
	Prepare staff to address common questions, e.g. "Why should I take praziquantel when I don't have symptoms?"
	Use mass media campaigns (radio, TV, traveling road shows, posters, booklets, brochures) for education of target populations
	Conduct education sessions in many venues, including village meetings, places of worship, markets, and other places where people gather
	Incorporate schistosomiasis/praziquantel education into school curricula
Reduce fear of side effects	Provide snacks with MDA distribution
	Schedule MDA when food is more plentiful (e.g. after a harvest)
	Educate community members about the range and transient nature of potential side effects
	Explain the link between worm burden and intensity of side effects and why side effects may thus be worse during the first round of treatment
	Have drug distributors and prominent community members publicly take praziquantel to demonstrate its safety
Motivate and retain drug distributors	Increase distributor-to-recipient ratios to reduce workload
	Provide small financial or material incentives
	Avoid scheduling schistosomiasis MDA concurrently with other health programs that provide incentives
	Avoid scheduling MDA during periods of the year with especially high agricultural or other demands

What the data say about how to achieve high coverage rates

- Combined delivery achieves the highest median coverage of school-aged children, followed by community-only delivery, then school-only delivery.
- WHO guidelines recommend at least 75% coverage, which was achieved by all studies using combined distribution but not by all studies using community-only or school-only distribution.
- Non-enrolled children had lower coverage overall compared to enrolled children, and school-based delivery had the lowest coverage of non-enrolled children compared to other delivery methods.

Overall, the best means to maximize coverage for both enrolled and non-enrolled children is to use a combined community- and school-based approach.

In order to better compare the efficacy of different MDA control strategies, more randomized control trials are needed, along with more consistent reporting of target population sizes and coverage rates.

This study provided a systematic look at how to design the most effective schistosomiasis MDA programs. In the push towards schistosomiasis elimination, consideration of these factors in the development of future control programs is essential.

Burnim M, Ivy JA, King CH (submitted for publication) Systematic review of effectiveness of community-based, school-based, and combined delivery modes in targeting school-aged children for mass drug administration in schistosomiasis control programs
