# Supplementary material for: The Schistosomiasis Consortium for Operational Research and Evaluation Rapid Answers Project: Systematic Reviews and Meta-Analysis to Provide Policy Recommendations Based on Available Evidence
Source: Am J Trop Med Hyg. 2020 May 12;103(1 Suppl):92–6. doi: 10.4269/ajtmh.19-0806 (PMC7351305; doi:10.4269/ajtmh.19-0806)
Supplement: Supplementary file 6 [file tpmd190806.SD6.pdf]

SCORE Rapid Answers Project (RAP):

The Decline in Infection-Related Morbidities Following Drug-Mediated Reduction in the Intensity of *Schistosoma* Infection

Background: Since 1984, WHO has endorsed drug treatment to reduce *Schistosoma* infection and its consequent morbidity. Cross-sectional studies suggest pre-treatment infection intensity correlates with risk for *Schistosoma*-related pathology. However, other evidence suggests that even if drug treatment reduces intensity, morbidity may not be reversed because some morbidities occur at all levels of infection, and some reflect permanent tissue damage. The aim of this project was to systematically review evidence on the impact of drug-based control on schistosomiasis-related morbidities, and to develop a quantitative estimate of this impact.[1]

Question: Does treatment of *Schistosoma* infection translate into reduced odds of infection-related morbidity? If so, by how much?

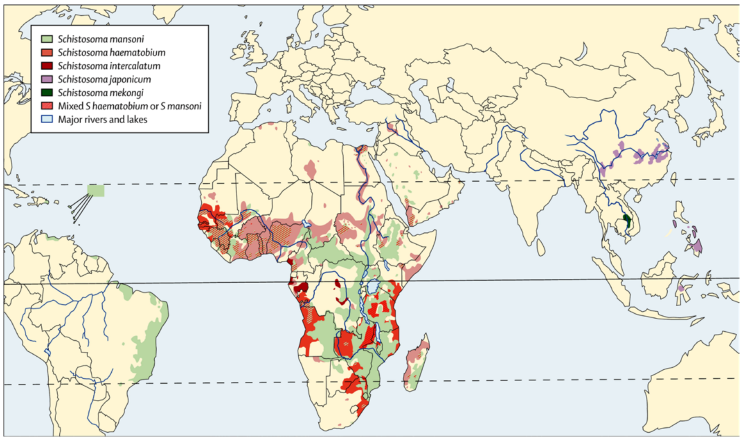

From: Colley, DG et al. (2014) Human Schistosomiasis, Lancet 383:2253-64.

Morbidities that Could be Evaluated by Meta-analysis

For <u>intestinal schistosomiasis</u> caused by *S. mansoni* or *S. japonicum*: blood in stool, diarrhea, periportal fibrosis, portal vein dilation, splenomegaly, right-sided hepatomegaly, left-sided hepatomegaly, anemia

For <u>urogenital schistosomiasis</u> caused by *S. haematobium*: upper urinary tract lesions, bladder lesions, proteinuria, hematuria, anemia

The Problem: Schistosomiasis is the disease caused by infection with *Schistosoma* parasitic flukes. Infection can cause anemia, diarrhea, abdominal pain, difficulties with learning, and decreased physical fitness. Depending on the infecting species, chronic *Schistosoma* infection can cause a variety of pathologies. *S. mansoni* and *S. japonicum* can cause liver and spleen enlargement, fibrosis and hypertension of the portal vein of the liver, and in some cases, death due to gastrointestinal bleeding. *S. haematobium* can cause bladder ulceration and kidney blockage, bladder cancer, and genital lesions that lead to fertility problems in both women and men.

Approach: In our study, we quantified the reductions in prevalence of infection-related morbidities among populations with *Schistosoma* infection, as achieved by giving one or more drug treatments. We systematically reviewed 71 available reports of *Schistosoma*-related morbidity reduction and conducted a meta-analysis of the available data to quantify the odds of persisting morbidity after treatment in relation to the egg reduction rate, ERR, a measure of how much egg counts change from pre- to post-treatment. A higher ERR indicates a greater impact on infection intensity with drug treatment.

Worldwide, schistosomiasis control is a constant challenge for public health services in endemic regions, mainly due to difficulties in preventing frequent reinfection during childhood and early adulthood. Chronic or recurrent infections lead to progressive inflammatory damage from parasite eggs that remain trapped in human tissues.

Key Findings

Our meta-regression indicates that post-treatment reductions in egg burden are significantly correlated with decreased morbidity. In particular, larger ERRs, which indicate acute reductions in worm burden, are associated with reversal of most acute pathology. More advanced chronic pathologies appear less responsive to single rounds of treatment, even with adequate ERRs, multiple rounds of treatment may be necessary to improve those outcomes. Factors affecting the magnitude of morbidity reductions include *Schistosoma* species, population studied, age and infection status of study participants, and how long after treatment follow-up occurred.

Schistosomiasis Consortium for Operational Research and Evaluation
CTEGD, University of Georgia, Athens, GA 30602
phone: 706-542-3334 • fax: 706-542-3582 • http://score.uga.edu

Morbidity Reduction

I. Examples of Treatment Impact

Relative reduction in odds of diseases after treatment of *S. haematobium*

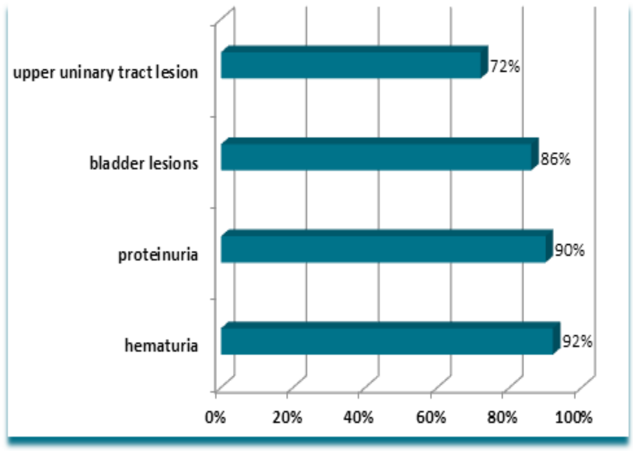

Relative reduction in odds of diseases after treatment of *S. mansoni or S. japonicum*

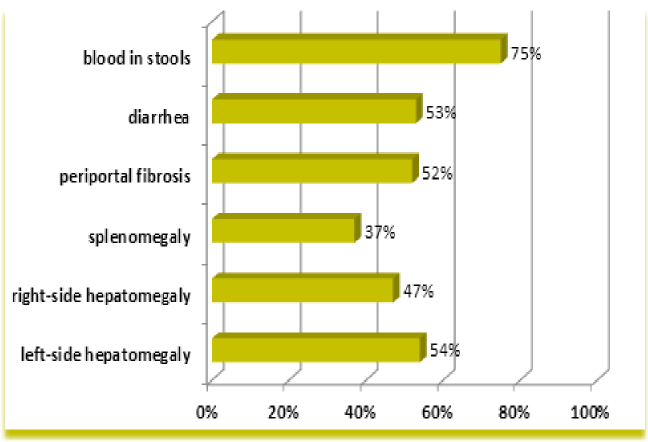

There were no consistent changes in portal dilation or blood hemoglobin levels.

II. Examples of Meta-Regressions Showing Relations Between ERRs and Odds of Diseases Post-Treatment

The graphs below plot the relative odds of disease post-treatment against ERRs. The higher the ERR, the greater the impact of treatment on infection intensity. These plots show that higher ERRs are associated with lower odds for post-treatment disease. Meta-regression lines (center black lines) and their 95% confidence limits (upper and lower blue lines) are shown for urinary tract bleeding when subjects had *S. haematobium* infections (left panel), and for periportal fibrosis of the liver when subjects had *S. mansoni* or *S. japonicum* infections (right panel).

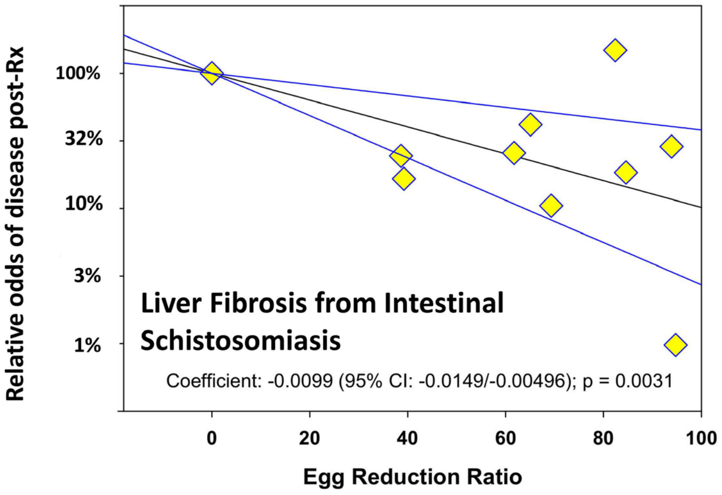

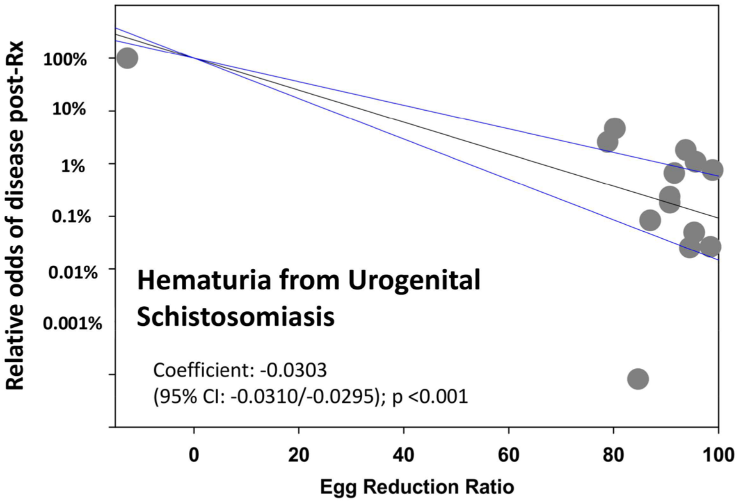

Implications

Our study shows that oral drug treatment reduces disease burden and supports continued efforts to reach populations at risk. Reducing all morbidity may require providing repeated treatment for people at risk for chronic and more intense infection. Because the reduction in egg output is significantly correlated with decreased morbidity, our estimates of the post-treatment odds of morbidity can be used to predict diminution in disease burden after successful program implementation. Nevertheless, our study was limited by gaps in the literature; additional well-designed and well-reported cohort studies are needed to strengthen the evidence base related to treatment impact on *Schistosoma* morbidity control.

[1]Andrade G, Bertsch DJ, Gazzinelli A, King CH (2017) Decline in infection-related morbidities following drug-mediated reductions in the intensity of *Schistosoma* infection: A systematic review and meta-analysis. PLoS Negl Trop Dis 11(2):e0005372. doi:10.1371/journal.pntd.0005372
