# Supplementary material for: The Schistosomiasis Consortium for Operational Research and Evaluation Rapid Answers Project: Systematic Reviews and Meta-Analysis to Provide Policy Recommendations Based on Available Evidence
Source: Am J Trop Med Hyg. 2020 May 12;103(1 Suppl):92–6. doi: 10.4269/ajtmh.19-0806 (PMC7351305; doi:10.4269/ajtmh.19-0806)
Supplement: Supplementary file 7 [file tpmd190806.SD7.pdf]

SCORE Rapid Answers Project (RAP):

Cognitive Deficits and Educational Loss in Children with Schistosome Infections

Summary: Empirical evidence for cognitive or education benefits of treating *Schistosoma* infection in children is limited. In a study completed in 2016, we addressed this knowledge gap by synthesizing information from 30 relevant epidemiologic studies reporting on 38,992 children between 5-19 years of age from 14 countries. In those studies, children with *Schistosoma* infection, or those who had not received treatment, were compared to unifected children or to children dewormed with praziquantel. Children with *Schistosoma* infection or who had not been de-wormed performed worse on psychometric tests of learning and memory. However, they performed similarly to the uninfected or dewormed children in tests of innate intelligence or reaction time. Infected or non-dewormed children had less school attendance and poorer scholastic achievement. Overall, the presence of *Schistosoma* infection or non-dewormed status was associated with educational, learning, and memory deficits in school-aged children. The combined evidence suggests that early treatment of children in *Schistosoma*-endemic regions could mitigate these deficits.[1]

Questions:

1. Among school-aged children examined in the context of cross sectional or case-control studies, is *Schistosoma* infection associated with worse performance in neurocognitive tests, or with educational loss?

2. Among school-aged children enrolled in prospective studies with specific treatment for *Schistosoma* infection, is lack of treatment with praziquantel associated with worse performance in neurocognitive tests, or with educational loss?

Key Finding:

Schistosoma infection/non-treatment was significantly associated with educational, learning, and memory deficits in school-aged children.

Pooled standardized mean difference (SMD) estimates of *Schistosoma* infection/non-treatment effects on educational/cognitive loss – with evaluation of study heterogeneity and publication bias

	# Studies	SMD (95%CI)[I]	Heterogeneity[Ψ] I^2	Publication bias P-value[α]
**Cognitive Domain**				
Memory	8	**-0.28 (-0.52, -0.04)**	78.6	0.786
Learning	6	**-0.39 (-0.70, -0.09)**	79.4	0.793
Intelligence Quotient Based Assessments	4	-0.25 (-0.57, 0.06)	74.8	0.450
Reaction Time	6	-0.06 (-0.42, 0.30)	88.5	0.142
**Educational Loss Assessments**				
Achievement	16	**-0.58 (-0.96, -0.20)**	97.9	0.595
School Attendance	16	**-0.36 (-0.60, -0.12)**	98.7	0.991

[I]Standardized Mean Difference (SMD) < 0 suggests a negative effect of infection/non-treatment on the indicated outcome; SMD > 0 indicates a positive effect of infection on respective outcomes. Bold font indicates statistically significant differences.
Ψ: measures the extent to which there is heterogeneity across studies in terms of underlying results.
α: evaluates the tendency for increased publication of studies that show a statistically robust finding; a $P < 0.05$ suggests presence of publication bias.

The findings of infection-related cognitive deficits and educational loss reported here are clinically relevant and should become an essential pillar in the design of schistosomiasis-related health policy. They reinforce the need to treat children with schistosomiasis early in life, so as to reduce their cumulative cognitive and functional morbidities.

Schistosomiasis Consortium for Operational Research and Evaluation
CTEGD, University of Georgia, Athens, GA 30602
phone: 706-542-3334 • fax: 706-542-3582 • http://score.uga.edu

Cognitive Deficits and Educational Loss

Examples of significant impact of *Schistosoma* infection - the gray boxes show estimates of SMDs and their 95% confidence intervals for individual studies that assessed learning and memory. Based on our meta-analysis, the blue diamonds indicate the overall summary estimates of the respective impacts of *Schistosoma* infection on measured learning and memory scores among affected children.

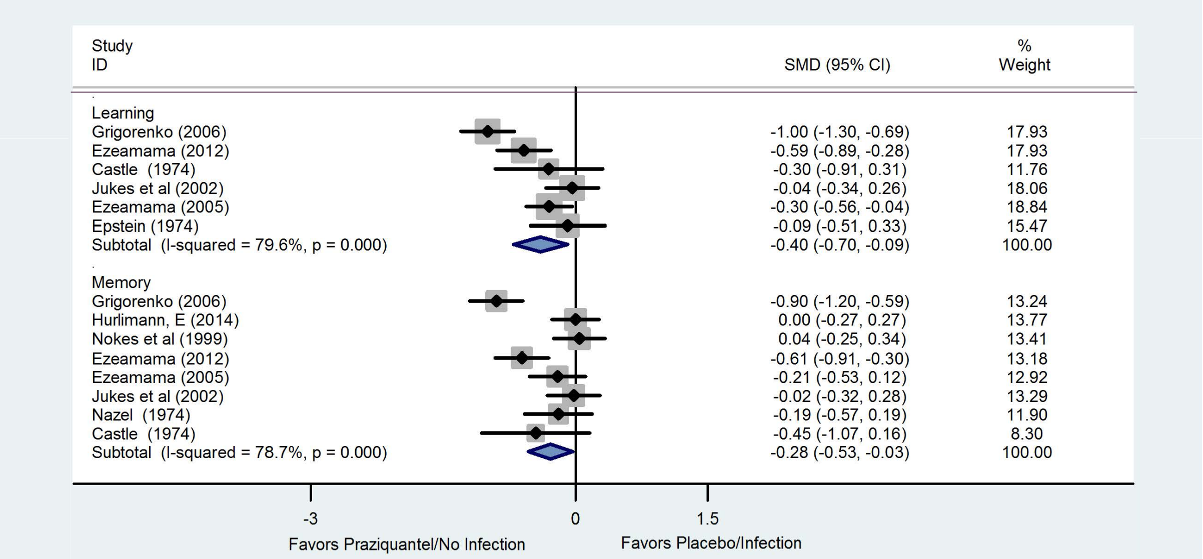

An estimated 800 million persons in tropical and sub-tropical countries are at risk of infection by one of three main human *Schistosoma* parasites - *S. mansoni*, *S. haematobium*, and *S. japonicum*.

Findings

- *Schistosoma* infection was associated with small-to-moderate deficits in psychometric tests of learning and memory.
- Infection was also associated with lower school attendance and scholastic achievement. Average effects on scholastic achievement were substantially larger for infection with *S. haematobium* than with *S. mansoni* infection.
- Deficits in learning and memory were clear both in analysis of observational studies and in longitudinal studies. Deficits related to educational achievement appeared robust among observational studies. They were less robust in the longitudinal studies, and risk of study bias was of potential concern.

The small-to-moderate deficits we observed at the individual level may amount to large and important differences in population achievement at the community level. It is not currently possible to estimate what the lifetime impact might be for individuals, as relatively small decrements in cognition or educational attainment in childhood may have larger impact on personal performance in later adult life.

Implications for school-aged children

Schistosoma infection often occurs in the context of malnutrition, coincident parasitic infections, and extreme poverty. Given the impacts of *Schistosoma* infection, increasing efforts to prevent or eliminate this disease are critical. If that is not yet possible, ensuring infected children receive treatment is important for their health and well-being. We hope that future studies of early childhood interventions, including treatment of schistosomiasis, will clarify to what extent the deficits we observed are reversible.

Implications for pre-school-aged children

Children from endemic areas are often infected by two years of age and remain chronically infected throughout their school-age years. These children may therefore suffer cumulative damage to their health and functioning that is currently not reflected in most short-term study outcomes. At present, there is no specific guidance for anti-*Schistosoma* drug treatment of pre-school children, partly because of the lack of a child-friendly pediatric formulation.

Given that we observed an adverse impact of *Schistosoma* infection on cognitive and educational domains in school-aged children, it is likely that the impact on younger children is at least as large or larger. Future longer-term studies should evaluate the impact of infection on pre-school children. These would provide important information for guiding decisions about preventive chemotherapy for and ensuring regular treatment of infected pre-school children.

[1]Ezeamama AE, Bustinduy AL, Nkwata AK, Martinez L, Pabalan N, Boivin MJ, et al. (2018) Cognitive deficits and educational loss in children with schistosome infection—A systematic review and meta-analysis. PLoS Negl Trop Dis 12(1): e0005524. https://doi.org/10.1371/journal.pntd.0005524
